# Design of a multi-epitope vaccine candidate against *Brucella melitensis*

**DOI:** 10.1038/s41598-022-14427-z

**Published:** 2022-06-16

**Authors:** Min Li, Yuejie Zhu, Ce Niu, Xinru Xie, Gulishati Haimiti, Wenhong Guo, Mingkai Yu, Zhiqiang Chen, Jianbing Ding, Fengbo Zhang

**Affiliations:** 1 The First Affiliated Hospital of Xinjiang Medical University, No. 393, Xinyi Road, Urumqi, 830011 Xinjiang China; 2 Department of Reproductive Assistance, Center for Reproductive Medicine, The First Affiliated Hospital of Xinjiang Medical University, No. 393, Xinyi Road, Urumqi, 830011 Xinjiang China; 3 Department of Clinical Laboratory, The First Affiliated Hospital of Xinjiang Medical University, No. 393, Xinyi Road, Urumqi, 830011 Xinjiang China; 4 Department of Immunology, School of Basic Medical Sciences, Xinjiang Medical University, Urumqi, 830011 China; 5 State Key Laboratory of Pathogenesis, Prevention, Treatment of Central Asian High Incidence Diseases, The First Affiliated Hospital of Xinjiang Medical University, No. 393, Xinyi Road, Urumqi, 830011 Xinjiang China

**Keywords:** Infection, Vaccines

## Abstract

Brucella is a typical facultative intracellular bacterium that can cause zoonotic infections. For Brucella, it is difficult to eliminate with current medical treatment. Therefore, a multi-epitope vaccine (MEV) should be designed to prevent Brucella infection. For this purpose, we applied the reverse vaccinology approach from Omp10, Omp25, Omp31 and BtpB. Finally, we obtained 13 cytotoxic T lymphocyte (CTL) epitopes, 17 helper T lymphocyte (HTL) epitopes, 9 linear B cell epitopes, and 2 conformational B cell epitopes for further study. To keep the protein folded normally, we linked AAY, GPGPG, and KK to CTL epitopes, HTL epitopes, and B cell epitopes, respectively. The N-terminal of the vaccine peptide is supplemented with appropriate adjuvants to enhance immunogenicity. To evaluate its immunogenicity, stability, safety, and feasibility, a final MEV containing 806 amino acids was constructed by linking linkers and adjuvants. In addition, molecular docking and molecular dynamics simulations were performed to verify the affinity and stability of the MEV-TLR4. Then, codon adaptation and in silico cloning studies were carried out to identify the possible codons for expressing the MEV. In animal experiments, the results demonstrated that the MEV had high immunogenicity. Collectively, this study provided a theoretical basis for the development of a Brucella vaccine.

## Introduction

Brucella is one of the Gram-negative bacteria that does not contain flagella and does not form spores or pods. The genus Brucella mainly includes 12 species, which are *Brucella melitensis*, *B. abortus*, *B. suis*, *B. ovis*, *B. canis*, *B. inopinata*, *B. neotomae*, *B. microti*, *B. vulpis*, *B. ceti*, *B. papionis*, and *B. pinnipedialis*. Thereinto, *B. melitensis* mainly infects goats and sheep, and human brucellosis is mostly caused by it^1,2^. The human brucellosis manifested as mild fever, itching rash, arthritis, fatigue, and muscle pain. And it is a systematic infectious disease that is transmitted to humans through ingestion of unpasteurized milk or via close contact with the primary host^3,4^. Brucellosis is a major human health problem in many countries, especially in Mediterranean areas, parts of South and Central America, and northern China^5^. Recently, it was reported that the incidence of brucellosis in China has risen dramatically^6^. However, there are still many clinical challenges in the diagnosis and treatment of brucellosis. This is because it has no specific clinical characteristics and grows slowly in blood culture. In addition, the disease is easy to develop into chronic, which can affect multiple organs at the same time. Therefore, it is necessary to develop a new therapeutic approach against Brucella in early prevention.

As biological products, vaccines can stimulate and induce specific immunity to specific pathogens, which are an effective way to prevent brucellosis^7^. Currently, vaccine research consists of many approaches, such as multi-epitope vaccines (MEV), vectored vaccines, DNA vaccines, and reverse genetics-engineered live-attenuated vaccines. MEV is an emerging vaccine development approach, which uses reverse vaccinology to improve the safety and efficacy of vaccine^8^. In the design of MEVs, it is necessary to select the appropriate protein as antigen. In previous studies, a series of outer membrane proteins and effector proteins were used to identify immunodominant antigens against *Brucella* spp. Outer membrane protein 10 (Omp10), one of the outer membrane proteins of *B. melitensis*, has been proved to have good immunogenicity in previous experiments and is suitable as a vaccine design candidate protein^9^. Outer membrane protein 25 (Omp25) is one of the outer membrane proteins of *B. melitensis* and has good immunogenicity. The difference is that Omp25 leads to the occurrence of an inflammatory response and the chronicization of brucellosis by activating the MAPK signaling pathway^10^. Therefore, based on Omp25, the vaccine may slow down disease progression. Similarly, outer membrane protein 31 (Omp31), the major outer membrane protein of *B. melitensis*, has been shown to have good immunogenicity in animal models of infection and is also a good choice for vaccine design^11,12^. BtpB, an effector protein of *B. melitensis*, is present in all species of the genus Brucella. It mainly reacts with MyD88, inhibits TLR signaling, and disrupts activation of dendritic cells BtpB, restraining TLR2, TLR4, TLR5, and TLR9 signaling. And it is also a new option for designing vaccines^13^. In summary, the four Brucella-associated proteins are suitable for the MEV design against *B. melitensis*.

In our study, we analyzed epitopes of Omp10, Omp25, Omp31, and BtpB, using diverse bioinformatics methods which were IEDB, NetCTLpan1.1, NetMHCIIpan4.0, ABCpred, and BCPREDS. Linkers are required for epitope linkage. The B-cell epitope and CTL epitopes were linked with the AAY linker, and HTL epitopes were linked together and to the CTL epitopes with the GPGPG linker^14^. However, epitope-linked vaccine proteins alone are susceptible to enzymatic degradation, so specific adjuvants were selected to stabilize the vaccine construct^15^. Then, we analyzed the physicochemical properties of the MEV, constructed its tertiary structure, and performed the molecular dynamics of the MEV-TLR4. Finally, animal experiments were carried out to validate the prediction results. The current study can provide theory and data for further research, and also can increase a new method and idea for the development of an MEV against *B. melitensis*.

## Materials and methods

### Selection of target proteins

ProtParam (http://web.expasy.org/protparam/) software was applied to analyze the physicochemical properties of proteins and MEVs. The physicochemical properties include the number of amino acids, molecular formula, acidity and basicity of amino acids, and a grand average of hydropathicity (GRAVY). VaxiJen (http://www.ddg-pharmfac.net/vaxijen/VaxiJen/VaxiJen.html) was applied to analyze the antigenicity of the proteins^16^. All outer membrane proteins and effector proteins of *B. melitensis* should be taken into account. However, based on existing studies, we compared the homology, antigenicity, hydrophilicity, and stability of some important outer membrane proteins and effector proteins. Finally, we found that these four proteins had good antigenicity, hydrophilicity, and stability in designing an MEV.

### Sequence retrieval

The amino acid sequences of Omp10 (Serial No.P0A3N8), Omp25 (Serial No.Q45321), Omp31 (Serial No.E0A8N6), and BtpB (Serial No.A0A1S6ZGG9) were obtained by applying the Uniprot database (https://www.uniprot.org/). Finally, we analyzed the homology of the obtained sequences by comparing the amino acid sequence using MAFFT in Jalview^17^.

### Prediction of target proteins

#### Prediction of signal peptide

SignalP5.0 (https://services.healthtech.dtu.dk/service.php?SignalP-5.0) and LiPOP1.0 (https://services.healthtech.dtu.dk/service.php?LipoP-1.0) were applied to predict the signal peptide of the proteins. Based on the results of the software, we choose the merged set as the final result of the prediction^18^.

#### Prediction of T-cell epitopes of proteins

T-cell epitopes are provided by class I (MHC I) and II (MHC II) MHC molecules that are recognized by two distinct subsets of T-cells, CD8, and CD4 T-cells, respectively^19,20^. Depending on the polymorphism of the HLA allele, the T cell response is different. We selected the alleles with high frequency in Xinjiang (HLA-A*1101, HLA-A*0201, HLA-A*0301, HLA-DRB1*0701, HLA-DRB1*1501, and HLA-DRB1*0301) to predict the cytotoxic T lymphocyte (CTL) epitopes and helper T lymphocyte (HTL) epitopes^21^. IEDB (http://tools.immuneepitope.org/) and NetCTLpan1.1 server (https://services.healthtech.dtu.dk/service.php?NetCTLpan-1.1) were applied to predict the CTL epitopes of the target protein^22,23^. During CTL epitope prediction 1 was set as a threshold value for epitope identification. Since NetCTLpan1.1 counts from 0, we add one to the starting sequence number of all NetCTLpan1.1 to facilitate comparison with the results of IEDB. Accordingly, we listed the top 10 high-score epitopes for each prediction software. IEDB and NetMHC-IIpan-4.0 (https://services.healthtech.dtu.dk/service.php?NetMHCIIpan-4.0) were applied to predict the HTL epitopes of the target protein^24^. When predicting HTL epitopes, the default threshold for NetMHC-IIpan4.0 is set to the original threshold.

Finally, we selected two software overlapping sequences as T cell dominant epitopes for the proteins.

#### Prediction of B-cell epitopes of proteins

B-cell epitopes are the antigen portion binding to the immunoglobulin or antibody. B-cell epitopes include linear epitopes and conformational epitopes. BCPREDS (http://ailab.ist.psu.edu/bcpred/predict.html) was used to predict the selection of dominant B-cell linear epitopes with a specificity of 75%. Then, discontinuous (conformational) epitopes were performed by Ellipro (http://tools.iedb.org/ellipro/) of the IEDB. After prediction, we also deleted the sequences with less than 5 amino acids and selected the sequences with high scores and appropriate lengths. In the end, linear epitopes and conformational epitopes are considered for the design of MEV.

#### The interaction between HLA alleles and T-cell epitopes: molecular docking

As common interactions of the HLA alleles, HLA class I (HLA-A*02:01) and HLA class II (HLA-DRB1*01:01) were selected to look for structural associations between the HLA alleles and the T cell epitopes. The molecular docking was performed in the HDOCK server.

### Development of a novel MEV

Non-allergic and non-toxic epitopes were selected for the MEV. Then, the selected epitopes were joined together with appropriate linkers to construct the vaccine sequence^25^. Different linkers were used to link B-cell epitopes together, the KK linker was added; for linking HTL epitopes together, the GPGPG linker was appended; and concurrently, for connecting CTL epitopes, the AAY linker was used. Human β-defensin-3 sequence (Serial No. Q5U7J2) and PADRE sequences were linked with the help of EAAAK linkers at the N-terminus. Finally, a polyhistidine tag was added at the C terminus to obtain the complete vaccine protein sequence^26^.

### Evaluation of vaccine construct

Bioinformatics software was used to synthesize and analyze the properties of the MEV. First of all, ProtParam software was applied to analyze the physicochemical properties of the MEV. VaxiJen was applied to analyze the antigenicity with an accuracy of 70 to 89%. Then, SOLpro (http://scratch.proteomics.ics.uci.edu/) was used to predict the solubility of MEV. The server has an overall accuracy of 74.15% and the threshold is 0.5^27^. Finally, AllergenFP was used to analyze its allergenicity.

### Prediction of secondary and tertiary structure

To predict secondary structures of MEV, the SOPMA (http://npsa-pbil.ibcp.fr/cgibin/npsa_automat.pl?page=/NPSA/npsa_sopma.html) secondary structure prediction tool was used. RoseTTAFold uses a machine learning-based method and includes a three-track network to process sequence, the distance between atoms, and coordinate information to predict the tertiary structure of the MEV^28^.

### Assessment of model quality

The quality of the tertiary structure models of the MEV was evaluated using the SWISS-MODEL Structure Assessment (https://swissmodel.expasy.org/assess) and ProSA-web server (https://prosa.services.came.sbg.ac.at/prosa.php)^29^. The assessment of the model quality is based on the Z-score and the dotted structure in the Ramachandran plot.

### Molecular docking

When the MEV enters the organism, the body recognizes it as a synthetic antigen and activates the immune system^30^. Therefore, we performed molecular docking to show the binding between proteins and immune molecules. We use the HDOCK server (http://hdock.phys.hust.edu.cn/) to perform the molecular docking between the MEV and the TLR4 (PDB ID: 4G8A) immune receptor^31^. Key interacting residues were deduced from the protein–ligand interaction diagram generated by LigPlot+ in combination and their 3D structure was visualized by PyMOL.

### Immune simulation

The vaccine enters the body as an antigen and also causes a corresponding immune response. C-ImmSim (https://kraken.iac.rm.cnr.it/C-IMMSIM/) was applied to simulate the extent and type of immune responses induced by the MEV in humans^32^. In the population of the Xinjiang region, high-frequency alleles of HLA-A*1101, HLA-A*0201, HLA-B*5101, HLA-B*3501, HLA-DRB1*0701, and HLA-DRB1*1501 were selected for analysis^21^. Simultaneously, the server simulates three sections that represent three different anatomical regions in mammals, including the bone marrow, the thymus, and the lymph. And three different intervals were 1, 84 and 168^33^. Finally, the parameters were set to the default values of the software, the simulation parameter random seed was set to 12,345, the simulation volume was set to be 50, and the simulation steps were set to 1050.

### Molecular dynamics simulation

Molecular dynamics (MD) simulation studies of the MEV-TLR4 complex were performed in Gromacs 2021.3^34^. Being solvated in a rectangular box of TIP3P waters, the resulting systems were extended up to a minimum cutoff of 15 Å from the protein boundary. Being added to the protein surface, Cl− or Na+ ions were used to neutralize the total charges of the systems. Afterward, the Amber ff14SB force field was employed for the protein in all of the MD simulations^35^. Using combined steepest descent and conjugate gradient method, the initial structures were fully minimized. Under canonical ensemble for 0.2 ns, the systems were then gently annealed from 10 to 300 K with a weak restraint of 15 kcal/mol/Å. What’s more, 100 ps of density equilibration was performed under an isothermal-isobaric ensemble at a target temperature of 300 K and the target pressure of 1.0 atm using Langevin-thermostat and Berendsen barostat with a collision frequency of 0.002 ns and pressure-relaxation time of 0.001 ns. Finally, a productive MD run of 10,000 ps was performed for all the complex systems after proper minimizations and equilibrations.

### Optimization of codons and in silico cloning

The XHOI and BamHI restriction endonuclease sites were selected and the codons of the vaccine were analyzed and optimized using the JAVA codon adaptation tool (http://www.jcat.de/). The DNA sequence of the plasmid was obtained from the Plasmid Files, and pET-28a (+) was selected as the vector. Then SnapGene 5.3.2 was used to analyze the suitable endonuclease sites in the multiple cloning site (MCS) region, and the target gene sequence of the MEV was inserted into the plasmid to complete the silicon cloning. And the MEV was synthesized and purified by SynPeptide Co Ltd (Shanghai, China). Lastly, the MEV was detected as endotoxin-negative by Limulus amebocyte lysate (LAL) before use.

### Simulation polymerase chain reaction and agarose gel electrophoresis

We designed the primers in SnapGene 5.3.2 based on the Tm value and the length size. The length of primers is generally 15–30 bp, Tm value is chosen as 72 °C, the annealing temperature of the upstream and downstream is 1 °C, GC content of upstream and downstream is about 40–60%, and protection nucleobase is added at the 5' end. The designed primers were further confirmed in Primer-BLAST (https://www.ncbi.nlm.nih.gov/tools/primer-blast/index.cgi?LINK_LOC=BlastHome)^36^. Finally, the recombinant plasmid was simulated agarose gel electrophoresis in SnapGene 5.3.2.

### Experiment method

#### Animal

Twenty female BALB/c mice (6 weeks old) were obtained from the animal experiment center of Xinjiang Medical University. Mice were kept in standard conditions. Followed the animal experiment requirements and operating guidelines of the Animal Ethics Committee of First Affiliated Hospital of Xinjiang Medical University, the experiment was strictly actualized. This study was approved by the Animal Ethics Committee of First Affiliated Hospital of Xinjiang Medical University (approval number 20220309-27). The mice were randomly divided into two groups (N = 10 per group), the control group, and the MEV group. Mice were instilled intranasally (i.n, 15 μL per nostril) with PBS (Control group), and 30 μg multi-epitope vaccine (MEV group) respectively for 15 days. The mice were executed and the respiratory lymph nodes (head, neck, and lower respiratory lymph node) and spleen were removed under sterile conditions. Then, the homogenate was prepared for cell isolation. The trachea was cannulated, and the lungs were gently lavaged 3 times with 1 mL of sterile PBS to obtain bronchoalveolar lavage fluid. Using a Pasteur capillary pipette without anticoagulant, blood was collected from the retro-orbital sinus and placed in coagulation microtubes. Finally, the blood was centrifuged and the serum was collected.

#### ELISA

To evaluate the immunogenicity of MEV, antigen-specific IgG1 and IgG2a titers in the immunized mice were tested by enzyme-linked immunosorbent assay (ELISA). Firstly, 96-well polystyrene plates (Greinerbio-one, Frickenhausen, Germany) were coated with MEV (1 μg/mL) at 100 μL/well and incubated at 4 °C overnight. Secondly, the plate was rinsed with TBS-T (Tris-buffered saline, pH 7.4, containing 0.05% Tween-20) 4 times, followed by blocking with PBS containing 10% fetal bovine serum (FBS) at 37 °C for 2 h. Thirdly, 100 μl of mouse serum diluted at 1:100 was added to the well and incubated at room temperature for 2 h. Then, after rinsing, horseradish peroxidase-conjugated goat-anti-mouse IgG1 or IgG2a antibodies (BD Pharmingen, Santiago, USA) were added and further incubated at 37 °C for 1 h. Thereafter, a substrate solution (BD OptEiA, BD Pharmingen, Santiago, USA) was added to each well. The plate was subsequently incubated at room temperature for 10 min, and 20% H2SO4 was added to terminate the reaction. Finally, using a microplate reader (Bio-Tropsch Tek Instruments, Winooski, Vt., USA), the absorbance value was measured at a wavelength of 405 nm. Similarly, to measure secretory immunoglobulin A (sIgA), 96-well plates were coated with 0.25 g/well MEV overnight at 4 °C. The bronchoalveolar lavage fluid (1:5 dilution) was added in triplicate into wells of the plates and incubated for 1 h at 37 °C. Then wells were washed. Horseradish peroxidase-conjugated goat anti-mouse IgA (1: 1000 dilutions; KPL) was added and incubated for 1 h. Finally, the reaction is completed by adding substrate, etc. in the same way.

#### IFN-γ ELISPOT

To further evaluate the HTL response, IFN-γ levels in the immunized mice were analyzed by enzyme-linked immunospot assay (ELISPOT). First of all, ELISPOT plates were coated overnight with an anti-mouse IFN-γ capture antibody (BD PharMigen, San Diego, CA) diluted at 1:60 in PBS at 4 °C. One day later, the plate was washed four times with phosphate-buffered solution (PBST) and once with PBS. Thirdly, plates were blocked with 100 μL of RPMI-1640 medium containing 10% fetal calf serum for 1 h at 37 °C. Afterward, the respiratory lymph nodes cells and splenocytes were added in triplicate to wells at 1 × 10^5^ cells/well in a final volume of 100 μL. Fifthly, cells were stimulated with the heat-killed RB51 (10 μg/mL) (a source of Brucella protein without LPS), and synthetic Toxoplasma SAG4 peptide (10 μg/mL). ConA (10 μg/mL) at 50 μL/well and PBS at 50 μL/well were used as the positive and negative controls, respectively. After that, the plates were incubated for 24 h at 37 °C in an atmosphere containing 5% CO2. After washing, 50 μL of biotin-labeled secondary antibody (BD PharMigen, San Diego, CA) was added and the plates were incubated at room temperature for 2 h. And the plates were washed with PBST and 50 μL streptavidin alkaline phosphatase (Amersham Life Science, Australia) was added and incubated at room temperature for 2 h. By adding BCIP/NBT developer (Moss, Inc, Pasadena, MD, USA) to each well for incubation for 40 min at room temperature, the spots were developed. Finally, the plates were washed in water to stop the reaction and Spot forming units (SFU) in each well were counted using an ELISPOT Bio Reader-4000 (BIOSYS, GmbH, Germany). The results were expressed as IFN-γ spot forming cells (SFC) per million cells.

### Approval for animal experiments

The animal study was reviewed and approved by The Animal Experiment Medical Ethics Committee of the First Affiliated Hospital of Xinjiang Medical University. We confirmed that all experiments were performed in accordance with relevant named guidelines and regulations. We confirmed that the authors complied with the ARRIVE guidelines.

## Results

### Selection of target proteins

**Figure 1:**
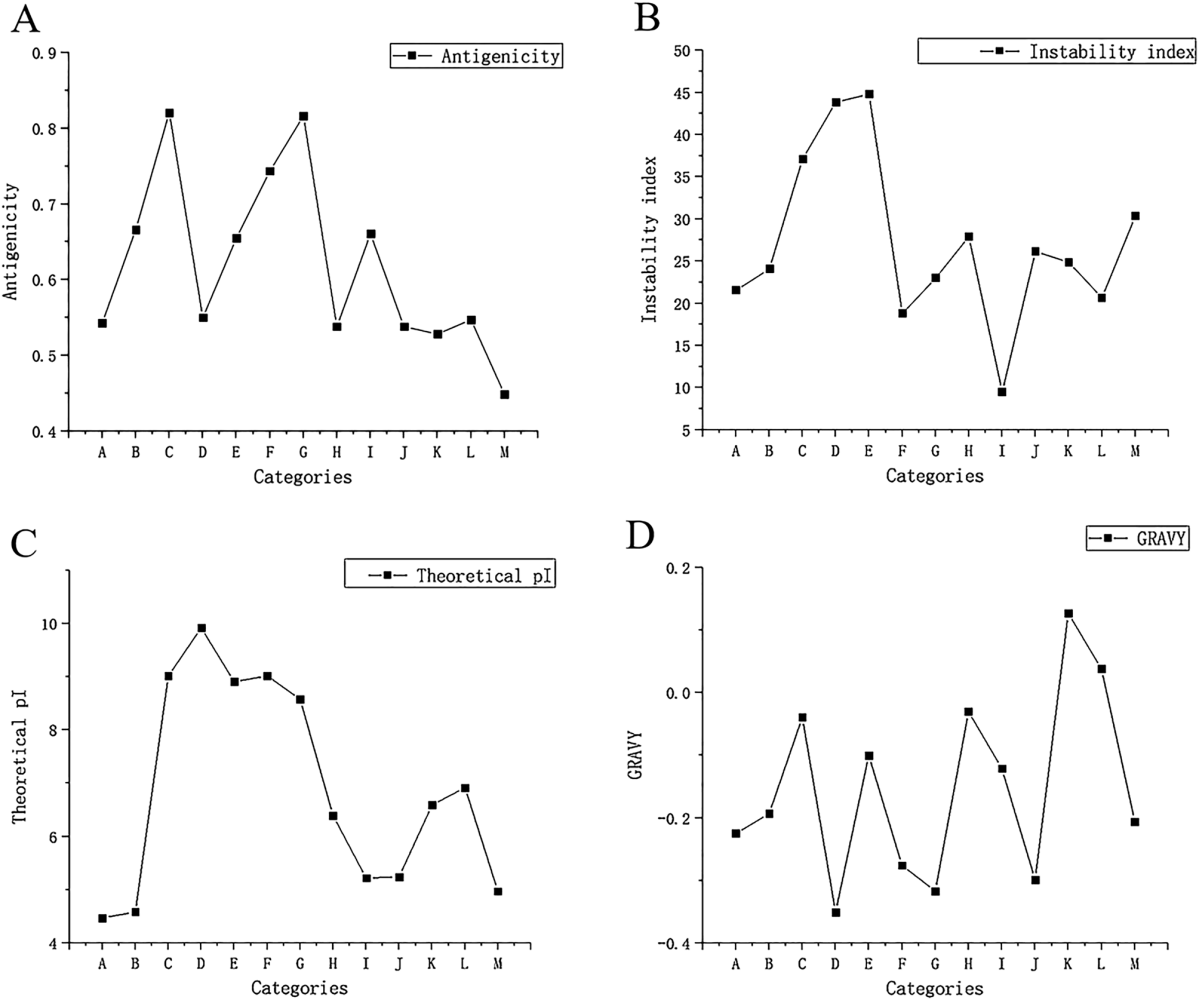
(**A**) Antigenicity prediction of 13 proteins. (**B)** Instability index prediction of 13 proteins. (**C**) Theoretical pi prediction of 13 proteins. (**D**) GRAVY prediction of 13 proteins. As illustrated in the figure, “A” stands for Omp2a, “B” stands for Omp2b, “C” stands for Omp10, “D” stands for Omp16, “E” stands for Omp19, “F” stands for Omp22, “G” stands for Omp25, “H” stands for Omp28, “I” stands for Omp31, “J” stands for BtpB, “K” stands for BLS, “L” stands for BCSP31, and “M” stands for P39.

The study found that the FASTA structures of protein from UniProt mainly include the following types: Omp2a (Accession: Q7CNU3), Omp2b (Accession: Q8YG56), Omp10 (Accession: P0A3N8), Omp16 (Accession: P0A3S7), Omp19 (Accession: P0A3P1), Omp22 (Accession: Q93SI4), Omp25 (Accession: Q45321), Omp28 (Accession: P0A3U8), Omp31(Accession: E0A8N6), BtpB (Accession: A0A1S6ZGG9), BLS (Accession: X5DV28), BCSP31 (Accession: P0A3T2), and P39 (Accession: F6N110). Through bioinformatics analysis, the antigenicity (Fig. 1A), instability index (Fig. 1B), theoretical pi (Fig. 1C), and GRAVY (Fig. 1D) of these proteins were compared. Finally, after comprehensive consideration, we selected the stable proteins with high antigenicity and hydrophilicity for the next step of the analysis.

### Sequence retrieval

**Figure 2:**
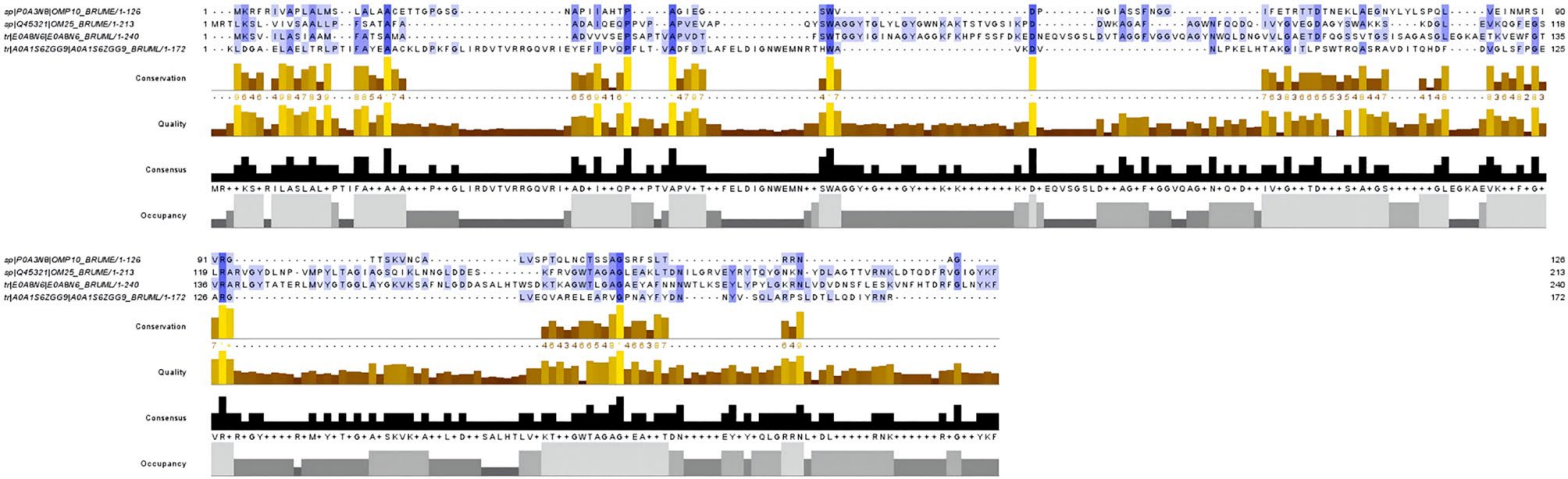
The homologous sequence comparison of the proteins. The black area in the figure is the highly conserved amino acid region, while the blue part is the similar amino acid sequence region.

The MAFFT in Jalview with the highest accuracy (Fig. 2) showed that these four proteins have several homologies. Thereinto, the high homology of Omp25 and Omp31 indicates that these proteins originated from the same gene during the evolution of *B. melitensis*, and suggests that they may play a similar role in the immune response.

### Prediction of target proteins

#### Prediction of signal peptide

**Figure 3:**
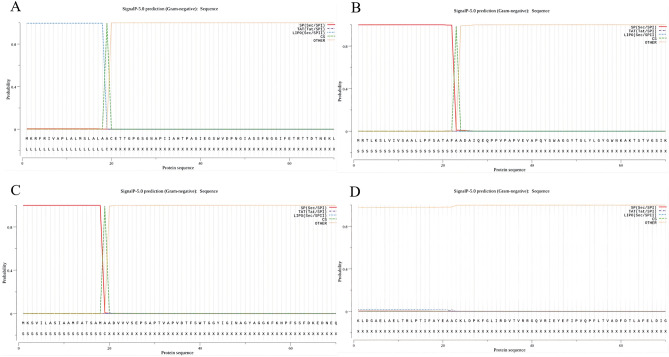
(**A–D**) Signal peptide of proteins using SignalP-5.0 analysis. SP (Sec/SPI): type of signal peptide predicted; CS: the cleavage site; Other: the probability that the sequence does not have any kind of signal peptide. (**A**) The signal peptide prediction of Omp10: MKRFRIVAPLALMSLALAAC. (**B**) The signal peptide prediction of Omp25: MRTLKSLVIVSAALLPFSATAFAA. (**C**) The signal peptide prediction of Omp31: MKSVILASIAAMFATSAMAA. (**D**) The signal peptide prediction of BtpB: none.

We applied two software to predict the signal peptide sequences of proteins. SignalP-5.0 (Fig. 3A) was used to predict the signal peptide of Omp10 (aa 1–20) and LiPOP1.0 was used to predict the signal peptide of Omp10 (aa 16–31). Therefore, we considered 1–31 peptide as the signal peptide of Omp10 with high probability. The result predicted by the former software is 1–24 peptide of Omp25 (Fig. 3B). And the result predicted by the latter software is 16–30 peptide of Omp25. Therefore, we deemed that 1–30 peptide is more likely to be the signal peptide. Similarly, the former software predicted 1–20 peptides of Omp31 (Fig. 3C), and the latter software predicted 14–31 peptides of Omp31. Therefore, we assumed that 1–31 peptides are more likely to be the signal peptide. Finally, BtpB (Fig. 3D) did not possess the signal peptide.

#### Prediction of T-cell epitopes of proteins

First of all, epitopes with high scores for each software were selected. Secondly, VaxiJen was applied to analyze the antigenicity of the epitopes. Furthermore, AllergenFP v1.0 (http://www.ddg-pharmfac.net/AllergenFP/) was used to detect whether epitopes are allergens. Then, the ToxinPred (https://webs.iiitd.edu.in/raghava/toxinpred/design.php) was applied to predict the toxicity of the epitopes^37^. Finally, 13 CTL dominant epitopes and 17 HTL dominant epitopes were obtained (Table 1). The antigenicity of these epitopes has higher antigenicity, and they were non-toxicity and non-allergenicity.

**Table 1:** List of the final selected CTL and HTL epitopes.

	Serials	CTL epitopes	Antigenicity	Serials	Th epitopes	Antigenicity
Omp10	49–58	GIASSFNGGI	0.5917	71–85	AEGNYLYLSPQLVEI	0.8234
	69–78	KLAEGNYLYL	0.7994	72–86	EGNYLYLSPQLVEIN	0.8829
	113–122	SAGSRFSLTR	1.0594	80–94	PQLVEINMRSIVRGT	0.4392
	114–123	AGSRFSLTRR	0.7167	84–98	EINMRSIVRGTTSKV	0.8259
				106–120	TQLNCTSSAGSRFSL	1.5871
Omp25	135–144	TAGIAGSQIK	1.4365	80–94	AGWNFQQDQIVYGVE	0.705
	168–177	KLTDNILGRV	0.7714	116–130	EGSLRARVGYDLNPV	1.2064
				119–133	LRARVGYDLNPVMPY	0.9872
				122–136	RVGYDLNPVMPYLTA	0.8388
				127–141	LNPVMPYLTAGIAGS	0.7646
				154–168	KFRVGWTAGAGLEAK	1.7619
				199–213	KLDTQDFRVGIGYKF	1.9370
Omp31	116–125	SAGASGLEGK	2.3499	89–103	GYNWQLDNGVVLGAE	0.9699
	139–148	RLGYTATERL	0.6381	137–151	RARLGYTATERLMVY	0.5659
	230–239	HTDRFGLNYK	1.3677			
BtpB	24–33	KLDPKFGLIR	1.3342	27–41	PKFGLIRDVTVRRGQ	0.7579
	54–63	FLTVADFDTL	0.4413	43–57	RIEYEFIPVQPFLTV	1.4844
	65–74	FELDIGNWEM	1.0865	45–59	EYEFIPVQPFLTVAD	1.035
	79–88	WAVKDVNLPK	0.4657			

#### Prediction of B-cell epitopes of proteins

**Figure 4:**
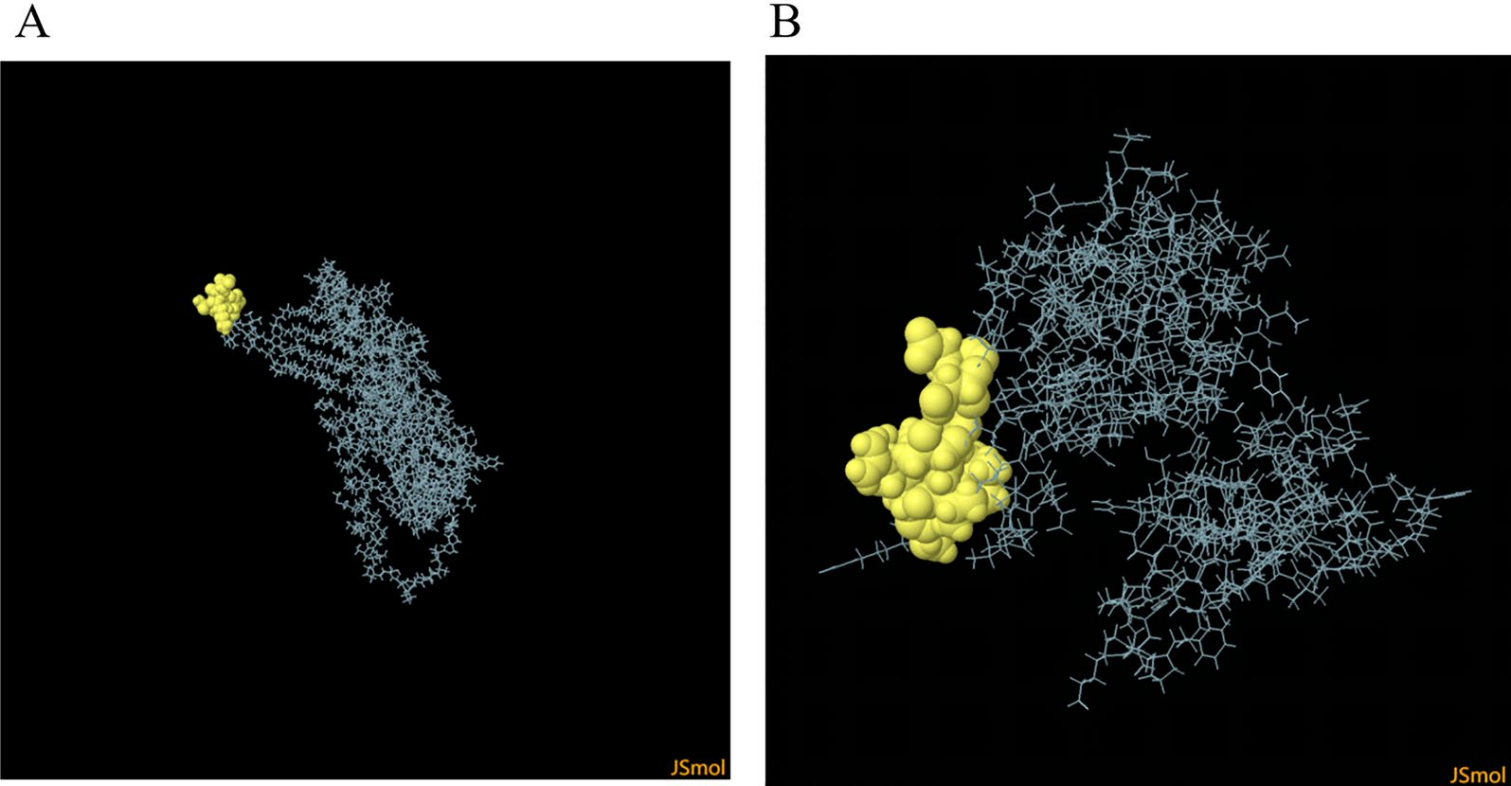
(**A**) ElliPro predicted the conformational B cell epitopes residues of Omp31: D64, K65, E66, D67, N68, E69. (**B**) ElliPro predicted the conformational B cell epitopes residues of BtpB: D34, T36, V37, R38, G40, Q41, V42, R43, E45.

Through bioinformatics analysis, we obtained 9 dominant linear B-cell epitopes. In the analysis of B-cell conformational epitopes, we obtained 5 conformational epitopes of Omp10, 4 conformational epitopes of Omp25, 8 conformational epitopes of Omp31, and 5 conformational epitopes of BtpB. Finally, we selected non-toxicity and non-allergenicity B cell conformational epitopes (Fig. 4) to construct the vaccine (Table 2).

**Table 2:** List of the final selected LB and CB epitopes.

	Serials	LBEs	Score	Antigenicity	CBEs	Score	After optimization	Serials
Omp10	52–71	SSFNGGIFETRTTDTNEKLA	0.957	0.8194	–	–	–	–
	100–119	CALVSPTQLNCTSSAGSRFS	0.786	1.156				
Omp25	89–108	IVYGVEGDAGYSWAKKSKDG	1	0.9693	–	–	–	–
	57–76	GWNKAKTSTVGSIKPDDWKA	0.899	0.4587				
Omp31	190–209	GAEYAFNNNWTLKSEYLYPY	1	0.9657	D64, K65, E66, D67, N68, E69	0.978	64–69	DKEDNE
	53–72	GGKFKHPFSSFDKEDNEQVS	0.997	0.4702				
	78–97	TAGGFVGGVQAGYNWQLDNG	0.991	0.9295				
	169–188	DDASALHTWSDKTKAGWTLG	0.837	0.6721				
BtpB	134–153	ARELEARVGPNAYFYDNNYV	0.872	0.766	D34, T36, V37, R38, G40, Q41, V42, R43, E45	0.839	34–45	DVTVRRGQVRIE

#### The interaction between HLA alleles and T-cell epitopes: molecular docking

**Figure 5:**
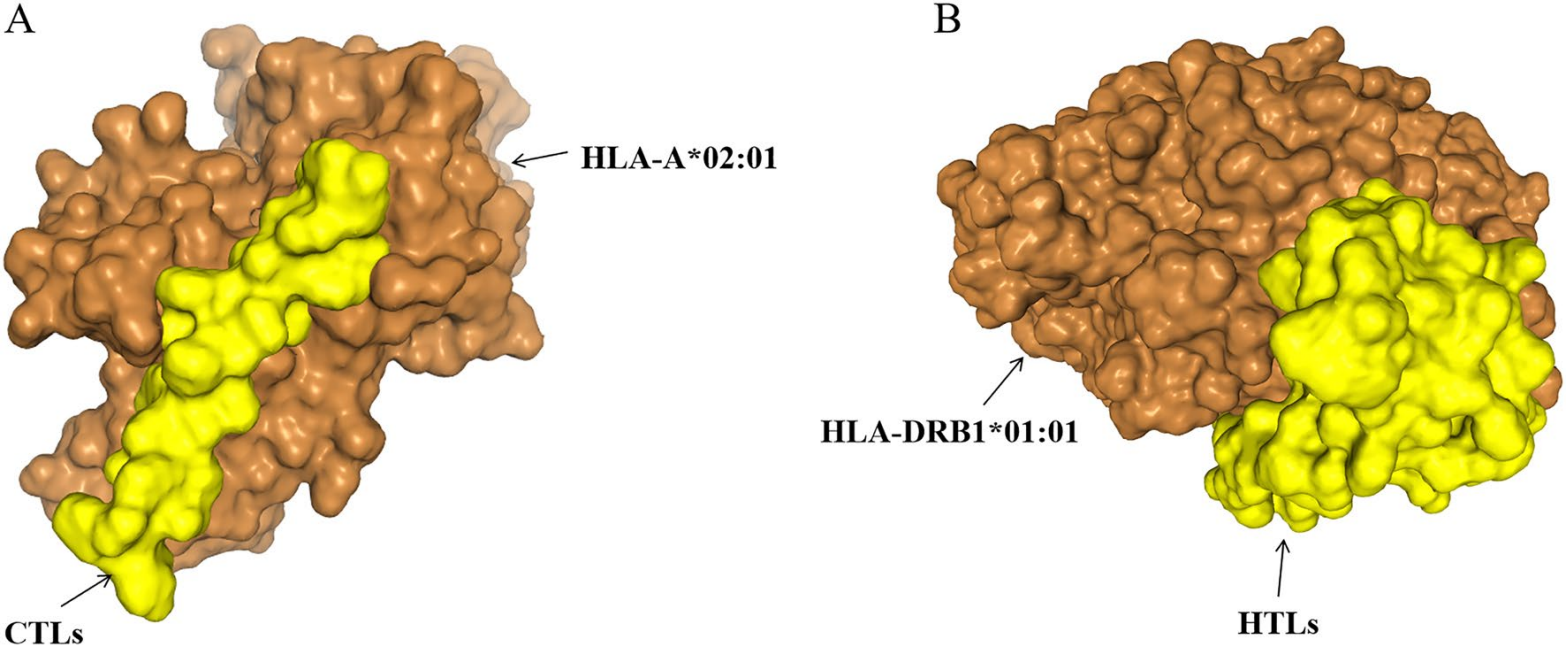
Screenshots of the HLA-bacterium peptide complexes. (**A**) HLA-A*02:01 (orange) and CTL epitopes (yellow) docking result. (**B**) HLA-DRB1*01:01 (orange) and HTL epitopes (yellow) docking result.

To evaluate the structural associations with HLA allele(s), we performed molecular docking simulations to find the interaction between the HLA alleles and the T cell epitopes. The interaction between HLA-I and CTL epitopes showed that the docking score was − 232.24 and ligand RMSD was 47.94 Å. The interaction between HLA-II and HTL epitopes showed that the docking score was − 326.30 and ligand RMSD was 143.19 Å. All the results indicated that docked complexes had a good affinity (Fig. 5).

### Development of a novel multi-epitope vaccine

**Figure 6:**
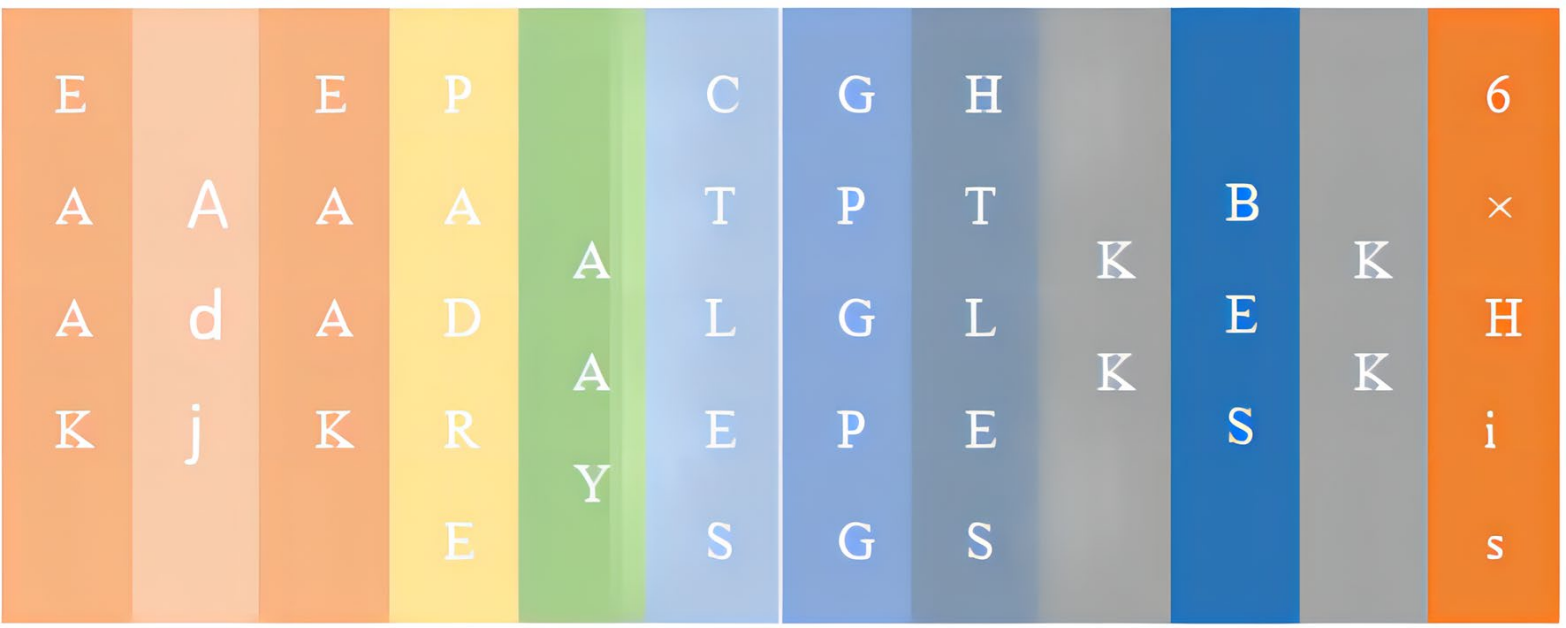
Schematic presentation of the final MEV. As illustrated in the figure, “Adj” stands for β-defensin-3, and “PADRE” stands for PADRE sequence. AAY, GPPGPG, and KK are linkers. BEs include linear B-cell epitopes and constitutive B-cell epitopes.

The MEV construct contained the dominant epitopes. There were 13 CTL epitopes, 17 Th epitopes, 9 LB epitopes, and 2 CB epitopes in the vaccine. All the dominant epitopes for the construction of MEV were shown in Fig. 6. The number of vaccine residues of the MEV was 806 amino acids.

### Evaluation of vaccine construct

The MEV had 806 amino acids, the molecular weight was 86,249.70 KD and the molecular formula was C3880H5980N1082O1122S16. The instability index (II) was computed to be 20.79, so the MEV was classified as a stable protein. The GRAVY was − 0.459, so the MEV belonged to hydrophilic protein. In addition, it had an antigenicity of 0.9750 (greater than the threshold) and had no allergenicity. What’s more, the solubility of the MEV was 0.99, which means that the protein antigen was soluble. Overall, the physicochemical properties of the MEV can be designed to meet our requirements.

### Prediction of secondary and tertiary structure

**Figure 7:**
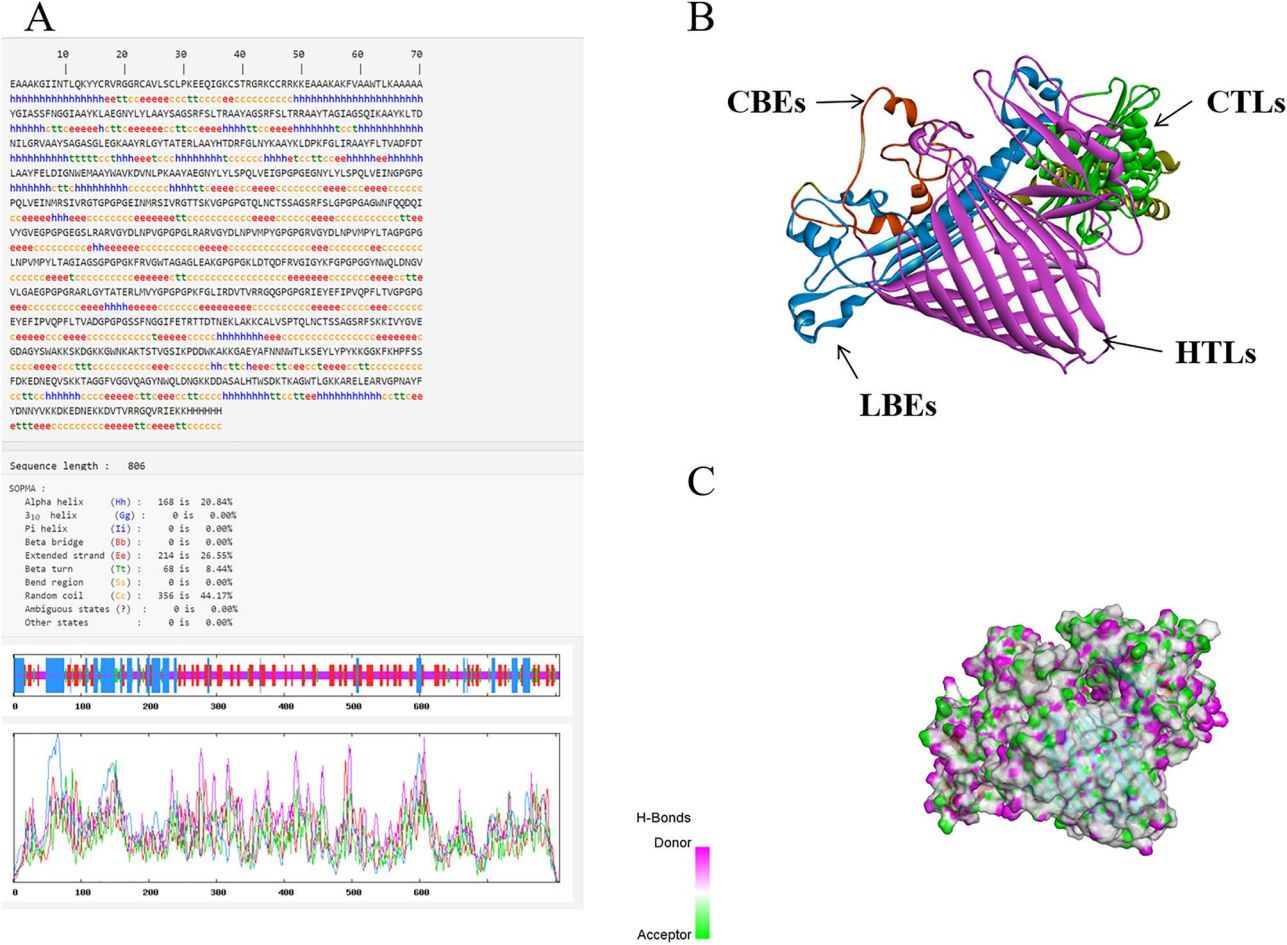
(**A**) The prediction of MEV secondary structure. (**B**) The prediction of MEV tertiary structure. (**C**) H-Bonds of MEV. As illustrated in the figure, the “pink area” stands for the donor, and the “green area” stands for the acceptor.

In the result of secondary prediction, there were 20.84% alpha-helix, 8.44% β-turn, 44.17% random coil, and 26.55% extended strand. The proportion of alpha-helix, β-turn, random coil, and the extended strand was consistent with the tertiary structure. This indirectly indicated that the prediction of the tertiary structure was reasonable. Then, we showed the PDB format of the MEV in Discover Studio, where pink in the model is more likely to represent the donor and green to represent the receptor (Fig. 7).

### Assessment of model quality

**Figure 8:**
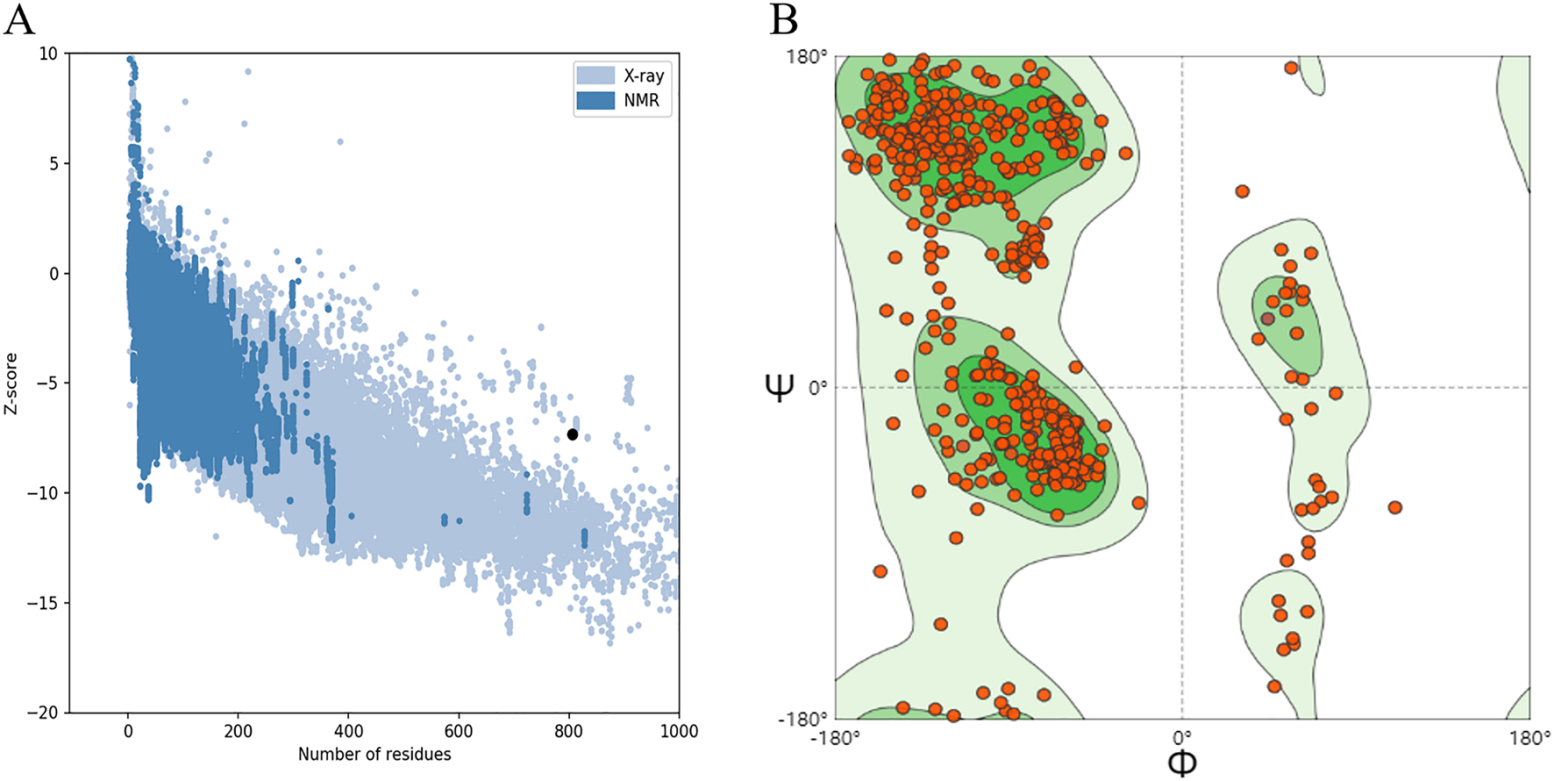
(**A**) Z-score plot obtained from ProSA-web. (**B**) Validation: Ramachandran plot analysis showing 90.80% in favored, 6.84% in allowed, and 2.36% in disallowed regions of protein residues.

ProSA-web was applied to assess the quality of the model, and the Z-score in the software indicated the quality of the model (Fig. 8). The light blue region indicated the structural group with the source of X-rays, and the dark blue region indicated the structural group with the source of nuclear magnetic resonance (NMR). The Z-score of MEV was − 7.32. The Z-score within the range characteristic for native proteins is indicative of a correct structure. To ensure the accuracy of the structure, we further evaluated the quality of the model using SWISS-MODEL’s structure evaluation service, which is called the Ramachandran plots. The dark green region in the Ramachandran plots indicated the allowed region, the light green region in the diagram indicated the maximum allowed region, and the blank region in the diagram indicated the disallowed region. It can be seen that most of the amino acids (i.e., dotted structures) fall almost completely inside the interval range. In conclusion, the tertiary structure of the MEV is the correct model with high confidence.

### Molecular docking

**Figure 9:**
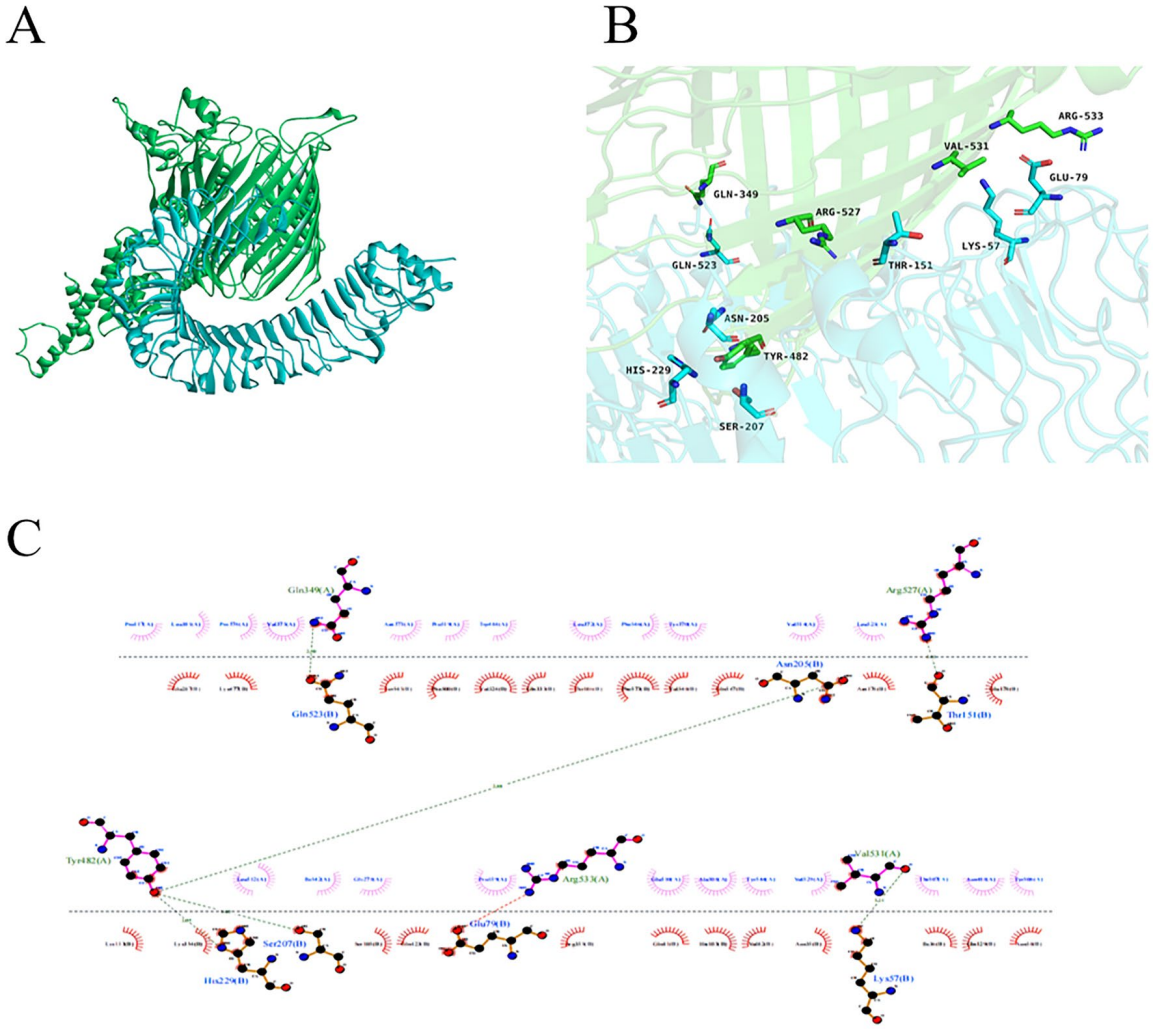
(**A**) Docked complex of vaccine constructs with TLR4; vaccine constructs in green color and TLR4 receptor in blue color. (**B**) The MEV-TLR4 complex was analyzed for interactions and their 3D images were taken by using visualizing tool PyMol. (**C**) The MEV-TLR4 complex was analyzed for interactions and their 2D images were taken by using visualizing tool Ligplot.

Molecular docking could effectively predict whether the ligand and the receptor could interact through the principle of energy minimization and the complementarity of the spatial structure in the region of the receptor active site. We obtained many clusters by molecular docking, of which we selected the number one cluster for presentation. The appropriate molecular docking results showed that the docking score was -328.00 and the ligand RMSD was 145.30 Å. After selecting the best docking structure (Fig. 9A), 3D structures were then visualized using PyMOL (Fig. 9B) and a 2D interaction map was visualized using LigPlot + (Fig. 9C)^38^.

### Immune simulation

**Figure 10:**
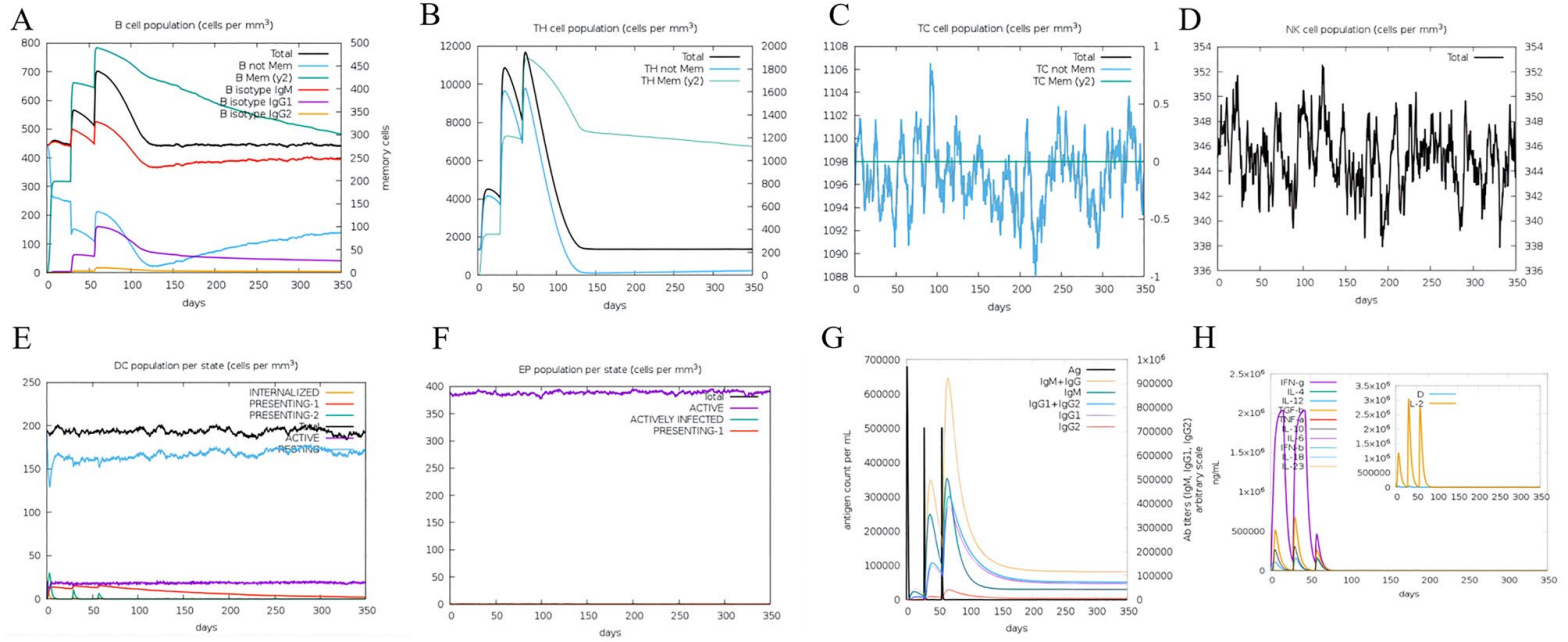
(**A**) The B-cell isotypes in various states. (**B**) The Th-cell isotypes in various states. (**C**) The TC-cell isotypes in various states. (**D**) The NK-cell in various states. (**E**) The DC-cell in various states. (**F**) The EP in various states. (**G**) The immunoglobulin in various states. (**H**) The interleukins and cytokines are in various states.

The C-ImmSim server was employed to predict the stimulated immune response against the MEV through a computational approach^32^. The simulator works by Miyazawa and Jernigan protein–protein potential measurements, applying input amino acid sequences to represent pathogen and lymphocyte receptors, and assessing the level of immune response elicited when the MEV is injected into the body. B cells are mainly involved in stimulating the immune response and cause an increase after each MEV injection. The number of B cells peaked in vivo after the last injection (Fig. 10A). Th cells and TC cells are important components of the T cell subpopulation. After three doses of the vaccine, the growth trend of Th cells (Fig. 10B) was less than that of B cells but reached a peak after three doses. In contrast, TC cells (Fig. 10C), NK cells (Fig. 10D), DC cells (Fig. 10E) and EP (Fig. 10F) generally remained relatively stable with dynamic changes. IgM and IgG concentrations are continually elevated in the primary and secondary immune responses accompanying antigen reduction (Fig. 10G). Finally, MEV can also elicit a highly responsive cytokine response (Fig. 10H), causing significant elevations in IFN-γ, TGF-β, IL-10 and IL-18.

### Molecular dynamics simulation

**Figure 11:**
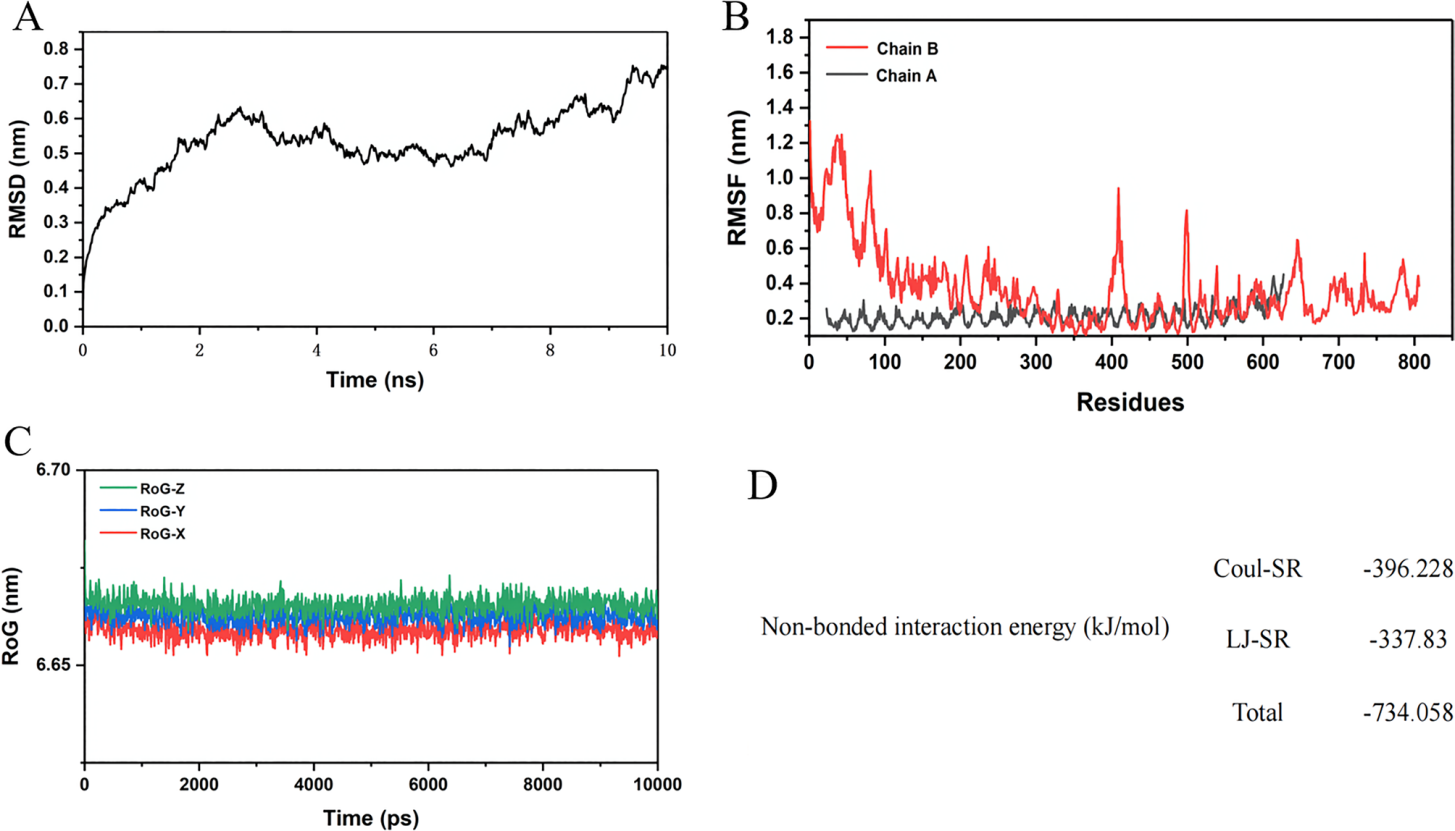
(**A–D**) The results of molecular dynamics simulation of the MEV-TLR4. (**A**) Root Mean Square Deviation analysis. (**B**) Root mean square fluctuation analysis. (**C**) The radius of gyration analysis. “Red” represents the X-axis direction, “blue” represents the Y-axis direction, and “green” represents the Z-axis direction. (**D**) Potential energy analysis that included Lennard–Jones short-range and Coulombic short-range.

MD simulations of the MEV with TLR4 structure need to be conducted. For this, a 10 ns simulation of the MEV-TLR4 was performed, using the GROMACS and MDrun tools. The structural parameters pertaining to the MD simulations like the root mean square deviations (RMSD), the root mean square fluctuations (RMSF), the radius of gyration (RoG), and the potential energy analysis of the MEV-TLR4 were estimated to assess their conformational and structural stability (Fig. 11). The structural variations of the MEV-TLR4 were examined by RMSD values from 0 to 10 ns, showing a steady increase at the beginning. After a relatively small range of fluctuations, the RMSD value reaches its highest value after 9 ns. The overall fluctuation range of the RMSD value does not exceed 0.65 nm. RMSF figure revealed that residues of the vaccine construct have mild fluctuations, especially in residues 100–650, which indicates the stability and uninterrupted interactions between the receptor and the construct. The compactness of the complex structure was determined by enumerating the Rog data of the complexes from the X, Y, and Z-axis. And the fluctuation range was less than 0.05 nm, which indicated that the backbone atoms in the complex structure are relatively stable in terms of gyration. Non-bonded interaction energy was calculated that are two types of short-range potential: Lennard–Jones short-range (LJ-SR) and Coulombic short-range (Coul-SR) potential. We calculated the total sum of LJ-SR and Coul-SR potential of ligands. For 10 ns simulation, the total residue interaction energy result was − 734.058 kJ/mol. The obtained sum of LJ-SR and Coul-SR potential were in line with the experimental activity of each ligand.

### Optimization of codons and in silico cloning

**Figure 12:**
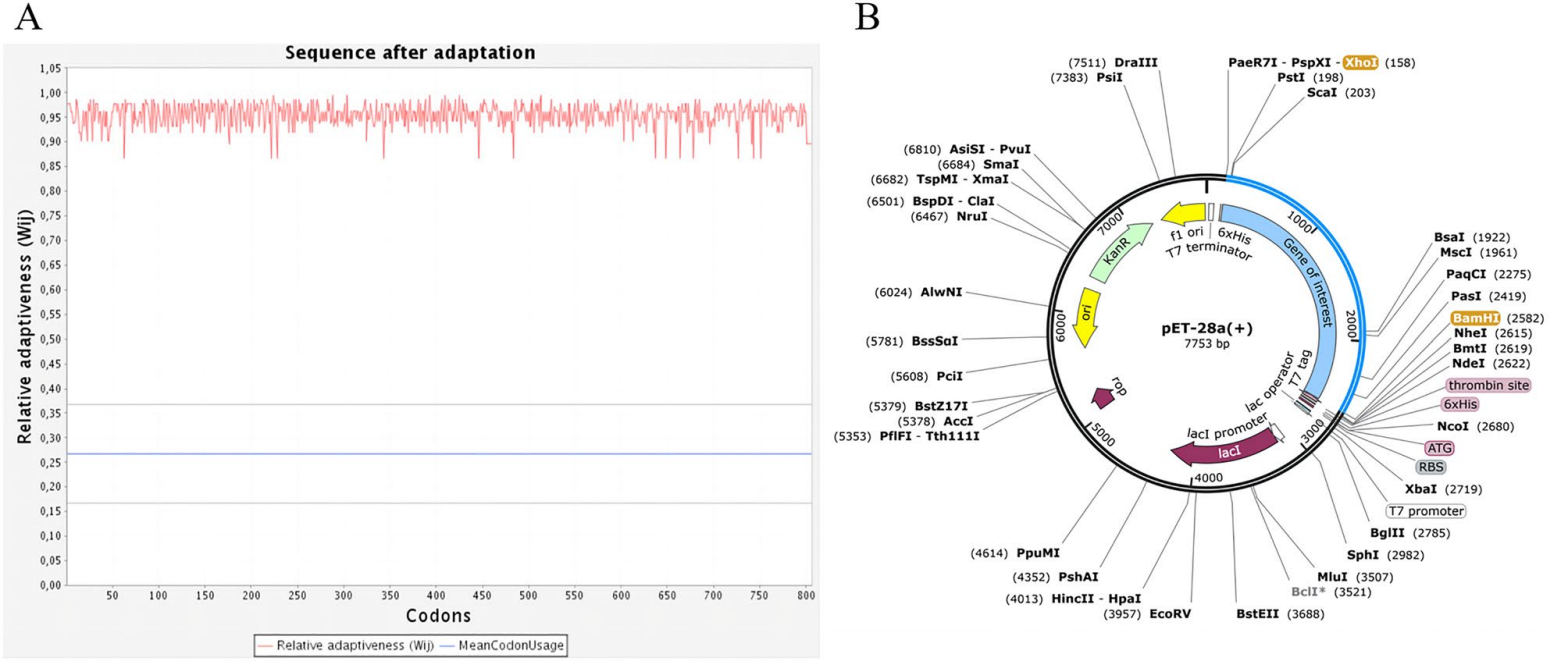
(**A**) Adaptation of optimized codons. (**B**) The MEV (blue) was inserted into the pET28a (+) vector (black) in silico cloning. The codon-optimized sequence of MEV (2418 bp) generated by the JCat server was inserted between XhoI (158) and BamHI (2582), forming a clone (7753 bp) using the SnapGene.

The codon adaptation index (CAI) is shown for coding sequences designed to maximize optimal codon usage (CAI = 1 indicates that the sequence was designed to have a codon adaptation index as close as possible to 1.00). The optimized CAI value of the MEV is 0.96. The ideal percentage range of GC content of the gene was between 30 and 70%, after optimization, the average GC content was changed to 70%. The BamHI and XHOI restriction enzyme sites were inserted at the N and C ends of the optimized codons, respectively. Then, the target gene was inserted into the MCS domain. Finally, specific restriction enzyme cutting sites were considered (Fig. 12).

### Simulation polymerase chain reaction and agarose gel electrophoresis

**Figure 13:**
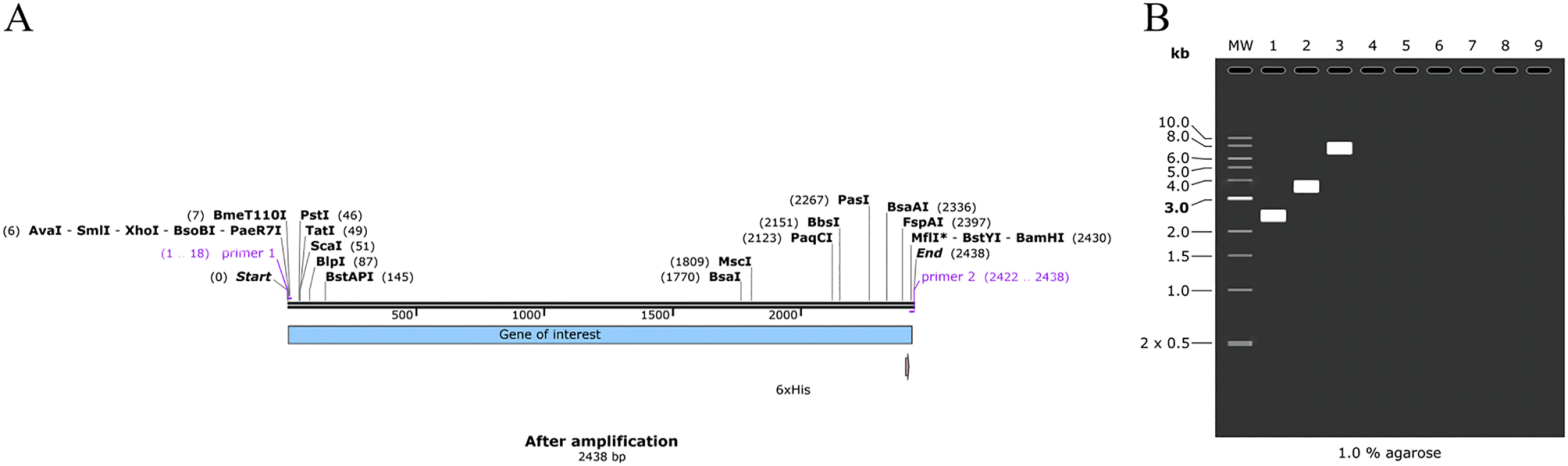
(**A**) The MEV (2438 bp) after amplified. (**B**) Mock agarose gel electrophoresis experiments. As illustrated in the figure, “1” stands for the MEV, “2” stands for pET-28a (+), and “3” stands for recombinant plasmid.

According to the above principles, we designed the forward primer (5′-CTTATCTCGAGGAGGCCG-3′) with a length of 18, Tm value of 56.31, and a GC content of 60%; the reverse primer (5′-CATGGATCCGTGGTGGT-3′) with a length of 17, Tm value of 56.05 and the GC content of 58%. BLAST results showed that the MEV shares the highest homology with the Homo sapiens zona pellucida glycoprotein 2 (ZP2). Afterward, the target gene of the MEV was amplified using SnapGene. We carried out the simulated agarose electrophoresis at a concentration of 1% and selected TBE with the better buffering ability of the target gene, vector, and recombinant plasmid in buffer solution. Finally, the amount of DNA was consistent with previous predictions. The sequence of the target gene was 2418 bp, the sequence of pET-28a (+) was 5639 bp, and the sequence of recombinant plasmid was 7753 bp (Fig. 13).

### The results of ELISA and ELISPOT experiments

**Figure 14:**
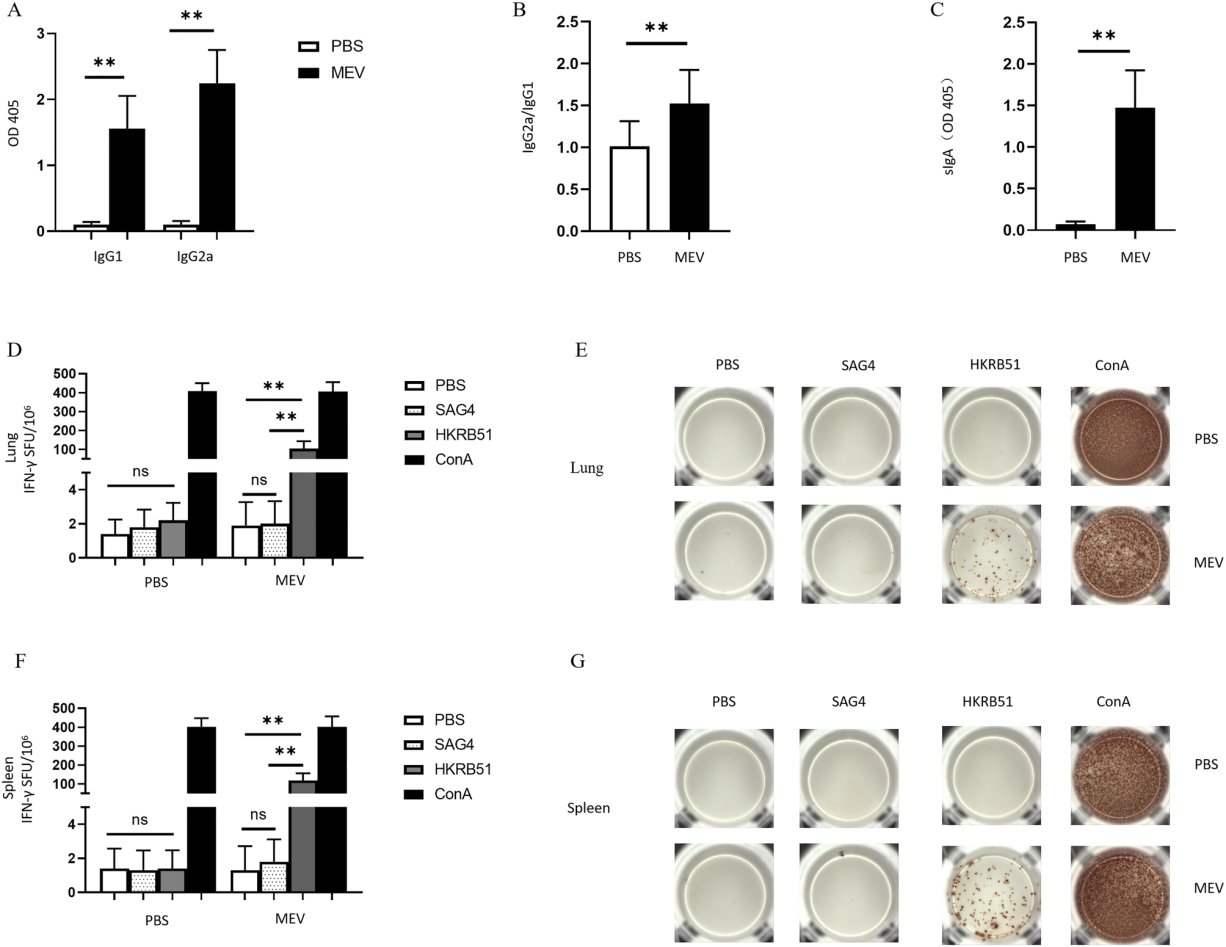
(**A**) The specific IgG1, and IgG2a antibodies in the immunized mice serum. The white part represents the control group and the black part represents the MEV group (P < 0.01). (**B**) The IgG2a/IgG1 ratio of the two groups (P < 0.01). (**C**) The level of sIgA in bronchoalveolar lavage fluid of the two groups (P < 0.01). (**D**) The number of T cells that can produce IFN-γ in respiratory lymph node specimens after antigen stimulation. (**E**) The representative ELISPOT spot diagram. SAG4 peptide and PBS were used as negative controls, ConA was used as a positive control, and HKRB51 was used as the experimental group. (**F**) The number of T cells that can produce IFN-γ in splenocytes specimens after antigen stimulation. (**G**) The representative ELISPOT spot diagram. One-way ANOVA was used for the Statistical Analysis. Data are shown as mean ± SD. P < 0.05 was considered statistically significant (**P < 0.01).

The MEV has to be tested in an animal model to see if the humoral and cellular immune response can be elicited. ELISA was used to verify whether humoral immunity could be produced. The results showed that the MEV group produced significantly higher levels of IgG1 and IgG2a (Fig. 14A) than the control group, which was dominated by the elevation of IgG2a (Fig. 14B). And the difference was statistically significant (P < 0.01). In the mucosal immune system, the results showed that the MEV group produced higher levels of sIgA (Fig. 14C) than the control group, and the difference was statistically significant (P < 0.01). In the same way, ELISPOT was used to verify whether cellular immunity could be produced. The results (Fig. 14D,F) showed that the MEV group produced significantly higher levels of IFN-γ than the control group, and the difference was statistically significant (P < 0.01). There were no statistically significant differences between the PBS group and the SAG4 peptide group (P > 0.05). The number of IFN-γ-producing cells (visualized as spots) can show the difference between results more visually (Fig. 14E,G). Our study demonstrated that MEV can elicit good humoral and cellular immunity in an animal model.

## Discussion

Brucellosis is a reemerged zoonotic infectious disease with complex clinical symptoms and high mortality rates^39^. The vaccine is still preferred for brucellosis prevention, but there are no existed Brucella vaccines for humans^40,41^. Several latest types of research have been reported recently, which encouraged the reverse vaccinology top down approach to get dominant epitopes from the whole proteome of viral and bacterial^42–44^. In our study, MEV was innovatively designed and its immunogenicity was evaluated in animal models.

Proteins with high antigenicity are possible targets for the construction of MEVs^45^. Therefore, based on the current research, we analyzed 13 major Brucella-associated proteins. After that, we chose Omp10, Omp25, Omp31, and BtpB with high antigenicity, stability, and hydrophilicity to build the MEV. At present, based on these four candidate proteins, there are no relevant reports for the construction of MEVs. In the current study, Omp25 of *B. melitensis* was shown to have significant homology with Omp31 of *B. melitensis*. At length, we again confirmed the high homology of these proteins when comparing the sequences, which provides a theoretical basis for future mechanisms of brucellosis pathogenesis^46^.

The signal peptide influenced the start of protein translation, and the different primary structures of the signal peptides even affected the folding and transport of the proteins^47^. Therefore, we removed the signal peptide sequences of Omp10 (1–31), Omp25 (1–30), and Omp31 (1–31), respectively. However, BtpB does not possess the signal peptide, so we considered the whole amino acid sequence can be further analyzed.

The objective of predicting HTL epitopes and CTL epitopes is to find the short peptide sequence within an antigen that stimulates CD4+ or CD8+ T cells in vivo^48^. Furthermore, the prediction of B cell epitopes of pathogenic organisms broadens the types of antigenic peptides and helps to improve the immunogenicity of antigens^49^. Therefore, four proteins were analyzed using IEDB and NetCTLpan1.1 server, which obtained 13 dominant CTL epitopes. Using IEDB and NetMHCIIpan4.0, 17 dominant HTL epitopes were acquired from four proteins. Using BCPREDS, 9 dominant linear B-cell epitopes were obtained from four proteins. Afterward, 2 dominant conformational B-cell epitopes from four proteins were obtained using IEDB. Ultimately, docked complexes between HLA alleles and T-cell epitopes had a good affinity.

It has shown that single-epitope-based vaccines had weak immunogenicity in the present study, but an MEV that consists of multiple epitopes can induce the activation of both cellular and humoral immune responses and enhance its immunogenicity^50^. Therefore, an effective MEV should be designed to include effective epitopes that can induce HTL, CTL, and B cells^51^. Three types of linkers that can keep proteins in are intrinsically ordered and normally folded states, including flexible linkers, rigid linkers, and cleavable (or self-cleavable) linkers^52,53^. The vaccine sequence has been constructed by combining CTL epitopes, HTL epitopes, and B-cell epitopes with the AAY, GPGPG, and KK linkers, respectively. After that, we constructed a vaccine with multiple epitopes. Adjuvants maintain the stability of the peptides and increase their immunogenicity^54^. There are several adjuvants used in reverse vaccinology studies, such as human β-defensin-3 (hBD3) and PADRE sequence^55,56^. HBD3 is a cationic peptide with immunomodulatory effects on both innate and acquired immune responses^55^. The PADRE sequence can reduce the effect of human HLA-DR polymorphism, and enhance the long-term immune response by inducing CD4+ T cells^57^. Finally, a complete MEV was obtained by adding adjuvants to the peptide sequence and adding the histidine sequence at the end.

In the early stages of vaccine design, some features of the MEV should be considered. In reverse vaccinology, theoretically, the molecular weight of the protein used to design the vaccine should be less than 110 KD^58^. The results of this project revealed that the MEV with 806 amino acids in length and molecular weight of 86 KD was a soluble protein. In addition, it had an antigenicity of 0.9750 (greater than the threshold) and had no allergenicity. These results indicated that the MEV had good stability, hydrophilicity, antigenicity, solubility, and non-allergenicity. After that, the prediction of the secondary structure showed that the β-turn and random coil accounted for 8.44% and 44.17%, respectively. The high proportion of β-turn and random coil in the MEV suggests that the protein is likely to form antigenic epitopes^59^. The RoseTTAFold had ultra-high tertiary structure prediction accuracy by successively integrating and converting the three-track network information of one-dimensional sequence, two-dimensional distance, and three-dimensional coordinates. Then, we used RoseTTAFold to predict the tertiary structures of four proteins and the MEV. Finally, the ProSA-web and the structure assessment service of SWISS-MODEL were used to verify the tertiary structure quality of the MEV. The results showed that the tertiary structure of MEV has high accuracy and a high approximation coefficient with the correct structure. In general, the tertiary structure of the MEV also exhibited a high number of β-turn and random coil, which is consistent with the results predicted by the secondary structure, indicating that the MEV has efficient antigen potential.

TLR4 is a transmembrane receptor protein with extracellular leucine-rich repeated domains and a cytoplasmic signaling domain, specifically recognizing endogenous molecules released from damaged or ischemic tissues termed pathogen-associated molecular patterns (PAMPs), and damage-associated molecular patterns (DAMPs)^60^. TLR4 recognizes lipopolysaccharides (LPS) of gram-negative bacteria and acts as a receptor for molecular docking with the MEV. Through docking, it can be seen that there is a strong interaction force between molecules through the atomic interaction interface between TLR4-MEV. The results suggest that the vaccine can develop stable connections with immune receptors so that it can be transported throughout the host body^61^.

To understand and explore the immune response profile and immunogenicity, an immune simulation study was conducted^62^. By carrying out the immune simulation experiment, we found that B and T cells in the body gradually increased after three doses of the vaccine and peaked at the time of the last dose of the vaccine. In addition, the MEV could also increase the levels of IFN-γ, TGF-β, IL-10 and IL-18. In particular, IFN-γ plays a major role in cell-mediated immunity by enhancing the bactericidal responses of macrophages, promoting the differentiation of Th1 cells, inducing B cell antibody class switching, enhancing cytotoxic responses of NK cells, and stimulating antigen presentation to T cells^63^. High levels of immune cells and cytokines indicate that the vaccine has great potential to induce a good immune response in humans. To further analyze the structural stability of the docked TLR4-MEV, a 10 ns MD simulation of the complex was performed. Taking into account the starting structure as reference for the TLR4-MEV, RMSD, RMSF, and ROG were computed to evaluate TLR4-MEV conformation changes and their stability. All the results suggested that the TLR4-MEV has strong stability.

The expression of the MEV in a suitable *E. coli* expression vector is pivotal for the production of recombinant proteins^64^. JCat was used for the optimization of codons, to increase the expression of MEV in the *E. coli*. CAI of optimized vaccine protein was 0.96 and the average GC content of our sequence was 70% showing good expression possibility of vaccine protein in *E. coli* host. After amplification, we obtained a 7753 bp recombinant plasmid that provided a favorable theoretical basis for further research.

To evaluate the effects of the MEV, we chose the specific antibodies as reliable indicators for humoral immunity and used IFN-γ as representative indices of cellular immunity. Higher IgG1, IgG2a, and sIgA demonstrated that MEV can elicit good humoral immunity in animal models. Simultaneously, higher levels of IFN-γ was demonstrated that MEV can elicit good cellular immunity in animal model.

Structure-based reverse vaccinology is an effective approach using rational vaccine design, but the resulting immune response does not represent effectiveness against all strains^65^. Because of this, we chose *B. melitensis* for further study, which is often infected by humans. In the present study, an MEV including CTL epitopes, HTL epitopes, and B cell epitopes, coupled with appropriate adjuvants, was designed to enhance the immune response in animal models. The results showed that this MEV becomes an excellent and suitable candidate against *B. melitensis*.

## Conclusion

Brucella is a gram-negative coccobacillus that can cause zoonotic diseases. Reverse vaccinology presented in the present work may produce new knowledge about vaccines against *B. melitensis.* Dominant epitopes were fused using linkers and adjuvant to enhance the MEV’s immunogenicity. Physiochemical properties, antigenicity, allergenicity, as well as solubility and tertiary structure analysis, of MEV were found to be very satisfactory. Moreover, the docked complexes during the simulation revealed that a strong and stable binding interaction of MEV with TLR4. In addition, this study used the C-ImmSim server to define the MEV, which is highly immunogenic. Finally, the study demonstrated that MEV can elicit both humoral and cellular immune responses in animal models.

## Data Availability

The data was derived from public domain information: Uniprot database (https://www.uniprot.org/) and PDB library (https://www.rcsb.org/). The data that support the findings of this study are available in the methods. The data that support the findings of this study are available from the corresponding author upon request. There are no restrictions on data availability. If you have any questions, please contact corresponding author.

## Abbreviations

MEV: Multi-epitope vaccine
Omp10: Outer membrane protein 10
Omp25: Outer membrane protein 25
Omp31: Outer membrane protein 31
CTL: Cytotoxic T lymphocyte
HTL: Helper T lymphocyte
TLRs: Toll-like receptors
TLR4: Toll-like receptor 4
LPS: Lipopolysaccharides
MD: Molecular dynamics
MAPK: Mitogen-activated protein kinase
RMSD: Root mean square deviations
RMSF: Root mean square fluctuations
RoG: The radius of gyration
LJ-SR: Lennard–Jones short-range
Coul-SR: COULOMBIC short-range
GRAVY: A grand average of hydropathicity
NMR: Nuclear magnetic resonance
hBD3: Human β-defensin-3
MCS: Multiple cloning site
CAI: Codon adaptation index
ELISA: Enzyme-linked immunosorbent assay
sIgA: Secretory immunoglobulin A
PAMPs: Pathogen-associated molecular patterns
DAMPs: Damage-associated molecular patterns
